# Structure of 2-chloro-*N*-(*p*-tol­yl)propanamide

**DOI:** 10.1107/S2056989018013889

**Published:** 2018-10-16

**Authors:** Roderick C. Jones, Brendan Twamley

**Affiliations:** a Synthesis and Solid State Pharmaceutical Centre (SSPC), School of Chemical and, Bioprocess Engineering, University College Dublin, Belfield, Dublin 4, Ireland; b School of Chemistry, Trinity College Dublin, University of Dublin, College Green, Dublin 2, Ireland

**Keywords:** crystal structure, API, continuous processing, biphasic

## Abstract

Two independent samples of the title compound, alternatively 2-chloro-*N*-(4-methylphenyl)prop­an­amide, C_10_H_12_ClNO, **1**, were studied using Cu *K*α, **1a**, and Mo *K*α, **1b**, radiation as part of a continuous crystallization study. The mol­ecule crystallizes with disorder in the Cl/terminal methyl positions [occupancies for the major disorder component of 0.783 (2) in **1a** and and 0.768 (2) in **1b**] and exhibits N—C bond lengths of 1.3448 (19), 1.344 (2) Å, C=O bond lengths of 1.2233 (18) and 1.2245 (19) Å and an acetamide moiety C—N—C—C torsion angle of 179.00 (13), 178.97 (14) ° for **1a** and **1b**, respectively. In the crystal, chains along the *a* axis are formed *via* N—H⋯O hydrogen bonds between acetamide groups, as well as C—H⋯O inter­actions. These chains arrange themselves into parallel running stacks which display weak C—Cl⋯O=C halogen bonding as well as weak C—H⋯π inter­actions.

## Abstract 2

Two independent samples of the title compound were studied using Cu *K*α and Mo *K*α radiation as part of a continuous crystallization study. In the crystal, chains along the *a* axis are formed *via* N—H⋯O hydrogen bonds between acetamide groups, as well as C—H⋯O inter­actions. These chains arrange themselves into parallel running stacks which display weak C—Cl⋯O=Chalogen bonding as well as weak C—H⋯π inter­actions.

## Chemical context   

**Figure 1:**
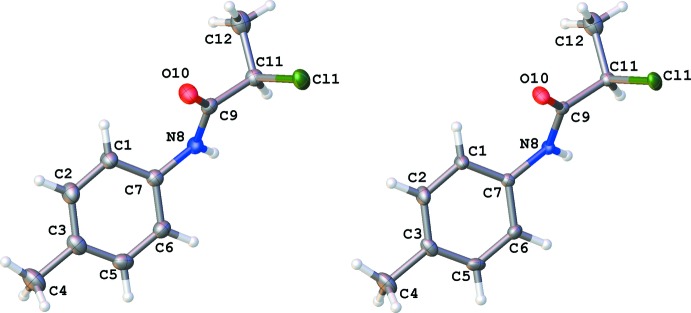
Mol­ecular structures **1a** and **1b** showing the atom-numbering scheme. Only the major occupancy disorder components [**1a** 0.793 (4) and **1b** 0.768 (2)] of the Cl1 and C12 positions are shown. Displacement ellipsoids drawn at the 50% probability level.

The introduction of continuous processing has been a paradigm shift in safety and productivity in the synthesis and isolation of active pharmaceutical ingredients (APIs) in both industry and academic research (Mascia *et al.*, 2013 ▸ and Lee *et al.*, 2015 ▸ and references contained therein). A major focus of our current research is developing design and optimization strategies to deliver robust, scalable and tunable continuous processes for API manufacturing, which can deliver specific API characteristics (Power *et al.*, 2015 ▸; Zhao *et al.*, 2015 ▸; O’Mahony *et al.*, 2017 ▸; Simon *et al.*, 2018 ▸). As part of this work we have been examining the continuous crystallization of 2-chloro-*N*-(*p*-tol­yl)propanamide, **1**, a key inter­mediate of α-thio-β-chloro­acryl­amides, a class of compound that has shown importance in the literature as synthetically viable APIs (Murphy *et al.*, 2007 ▸; Foley *et al.*, 2011 ▸; Kissane & Maguire, 2011 ▸) that can undergo transformations; such as Diels–Alder cyclo­additions (Kissane *et al.*, 2010*a* ▸), 1,3-dipolar cyclo­additions (Kissane *et al.*, 2010*b* ▸), sulfide group (Kissane *et al.*, 2010*c* ▸,*d* ▸) and nucleophilic substitution (Kissane *et al.*, 2011 ▸). To design and understand a continuous crystallization process for **1**, an extensive solubility study was conducted examining the compound’s solubility characteristics in common organic solvents (Pascual *et al.*, 2017 ▸). During this study, an improved bi-phasic synthesis was developed and crystals from two different continuous crystallization process runs were isolated to detect and characterize any variability of the crystalline material produced. These samples, **1a** and **1b**, of 2-chloro-*N*-(*p*-tol­yl)propanamide, see Fig. 1 ▸, are described herein.
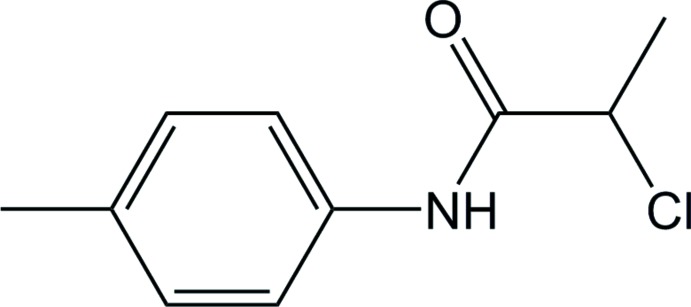


## Structural commentary   

Compound **1a** and **1b** both crystallize with one mol­ecule in the asymmetric unit in the ortho­rhom­bic space group *Pbca* and exhibit normal bond lengths and angles compared to similar compounds (2-chloro-*N*-phenyl­propanamide, IQOHOL, Gowda *et al.*, 2003 ▸ and references below). The disorder observed in **1** between the meth­yl/chloro positions is similarly displayed in IQOHOL. The aryl ring-to-amide backbone plane is twisted with a C1—C7—N8—C9 torsion angle of 45.3 (2) in **1a** and 45.6 (2)° in **1b** (Table 1 ▸).

**Figure 2:**
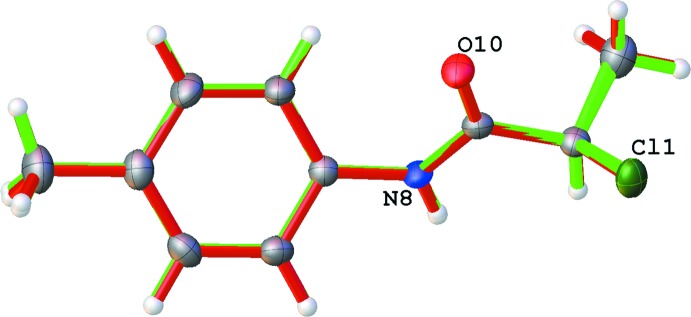
Overlay image of both mol­ecules of 2-chloro-*N*-(*p*-tol­yl)propanamide (**1a** is shown in red and **1b** in green) with an r.m.s. fit of 0.040 Å (no inversion). Displacement ellipsoids shown at the 50% probability level. Selected atom numbering only for clarity.

An overlay of the mol­ecular structures of **1a** and **1b** without inversion and an r.m.s. fit of 0.040 Å is shown in Fig. 2 ▸. The data, collected using different sources (Cu *K*α for **1a** and Mo *K*α for **1b**), show remarkable similarity even down to the hydrogen-bonding metrics seen in Tables 2 ▸ and 3 ▸. Data were collected on crystals of a similar size and at 100 K. As can be seen in Table 1 ▸, a comparison between several bond lengths and angles in **1a**, **1b** and IQOHOL show how the metrics are similar, even with data that was collected at room temperature (IQOHOL). The disorder occupancy is different in **1a**, **1b** and in IQOHOL, but to no great extent with the occupancy of the major component being 0.783 (2) for **1a**, 0.768 (2) for **1b** and for 0.899 IQOHOL.

## Supra­molecular features   

In the extended structure there is, as expected, a strong amide hydrogen bond, between the N—H group and the ketone oxygen (N8⋯O10^i^, see Tables 2 ▸ and 3 ▸). This feature can be seen in many of the known phenyl­acetamides and the donor–acceptor distance in similar congeners below range from 2.8175 (8) Å (XIHMOQ; Gowda *et al.*, 2001 ▸) to the longer inter­action in CEXPOK of 3.2576 (6) Å. The distance in IQOHOL is 2.8632 (6) Å, slightly longer than that found in **1**.

**Figure 3:**
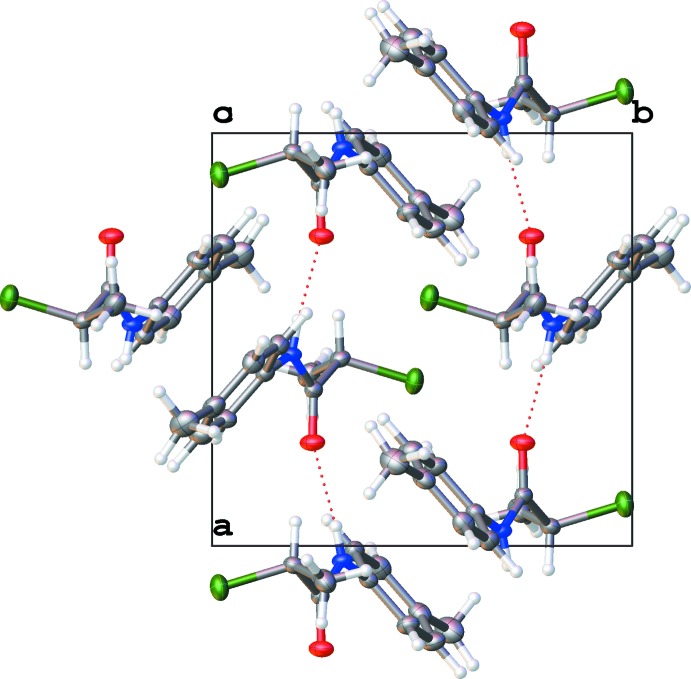
Hydrogen-bonding network represented by dotted lines of one layer in the cell viewed normal to the (001) plane. Displacement ellipsoids are shown at the 50% probability level.

There is also a weaker inter­action between the methine group and the ketone (C11—H11⋯O10^i^, see Tables 2 ▸ and 3 ▸). This type of chelate hydrogen bonding is also seen in IQOHOL and XIHMOQ [*D*⋯*A* = 3.2699 (8) and 2.8632 (6) Å respectively)] The head-to-tail packing and the chelate hydrogen bonding allows an approximately linear arrangement of **1**, forming ribbons propagating along the [100] direction, see Fig. 3 ▸. Only IQOHOL and XIHMOQ exhibit similar characteristics with head-to-tail and approximately linear packing [0.21367 (6) and 3.5472 (14)° respectively, as measured by the amide OCN and aryl carbon plane normal to plane normal angle, compared to 1.80942 (8)° in **1a** and 1.71940 (13)° in **1b**).

There are other supra­molecular inter­actions that assist in the packing of **1**. Complimenting the hydrogen bonding above, there is a weak C—Cl⋯O^ii^=C^ii^ halogen bond between the terminal chlorine and the ketone, with distances of **1a**, 3.1761 (14) and **1b**, 3.1734 (18) Å [symmetry code: (ii) 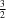 − *x*, −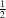 + *y*, *z*]. A very weak example of a C—H⋯π^iii^ inter­action is also present in **1**, with the methyl group C12 directed towards the centroid of ring C1–C6 (see Tables 2 ▸ and 3 ▸).

## Database survey   

A search of the Cambridge Structural Database (CSD version 5.39, February 2018 update; Groom *et al.*, 2016 ▸) for similar systems (*R*-PhNHCOCH–, where *R* = H, methyl, halogen) yielded several similar substituted phenyl­acetamides: CLACTN (Subramanian, 1966 ▸), CLACTN01 (Gowda *et al.*, 2007*a* ▸), CLACTN02 (Naumov *et al.*, 2007 ▸), CLACTN03 [Coles (née Huth) *et al.*, 2008 ▸], CEXPOK (Banks *et al.*, 1999 ▸), FOWYIA (Gowda *et al.*, 2009 ▸), IFALIK (Frohberg *et al.*, 2002 ▸), IQOHOL (Gowda *et al.*, 2003 ▸), JODQEZ (Si-shun Kang *et al.*, 2008 ▸), NIYYEB (Pathak *et al.*, 2014 ▸), NUWQUT (Hursthouse *et al.*, 2009 ▸), NUZBUF (Pal *et al.*, 1998 ▸), NUZBUF01 (Gowda *et al.*, 2001 ▸), RIYWIG (Gowda *et al.*, 2008 ▸), SALYIN (Chekhlov *et al.*, 1987 ▸), WINSUI (Gowda *et al.*, 2007*b* ▸), XEKNEJ (Gupta *et al.*, 2017 ▸), XICMAY (Gowda *et al.*, 2007*c* ▸), XIHMIK and XIHMOQ (Gowda *et al.*, 2001 ▸) and XIQNIV (Staples & Vidnovio, 2007 ▸).

## Synthesis and crystallization   

A solution of α-chloro­propionyl chloride (1.16 mL, 12mmol 1.2 equiv.) in toluene (30 mL) was added dropwise (with extreme caution) to a vigorously stirred bi-phasic suspension of *p*-toluidine (1.07 g, 10 mmol) in toluene (50 mL) and 40 mL of aqueous NaOH (1.20 g, 30 mmol, 3 equiv.) at 273 K. After the addition was complete, the biphasic suspension was warmed to room temperature and stirred vigorously for 1 h. The organic phase was separated, and the aqueous layer extracted with ethyl acetate (3 × 15 mL). The organic layers were then combined, dried with Na_2_SO_4_, filtered and the solvent removed under vacuum. The resulting off-white solid was collected and washed with thoroughly with cold cyclo­hexane (1.89 g, 96%). Single crystals for X-ray analysis were grown by slow evaporation of a toluene solution at room temperature. Spectroscopic data for the obtained product matched that reported in the literature (Pascual *et al.*, 2017 ▸).

^1^H NMR (300 MHz, CDCl_3_): δ 8.21 (*s*, 1H), 7.42 (*d*, *J* = 8.2 Hz, 2H), 7.15 (*d*, *J* = 8.2 Hz, 2H), 4.54 (*q*, *J* = 7.1 Hz, 1H) 2.13 (*s*, 3H), 1.83 (*d*, *J* = 7.1 Hz, 3H). ^13^C NMR (100 MHz, CDCl_3_): δ 166.9 134.4, 134.0, 129.1, 119.7, 55.9, 22.4, 20.5. MS (EI) *m*/*z* 197 [*M*]^+^, [^12^C_10_H_12_^35^Cl^14^N^16^O 197]. HRMS (EI) *m*/*z* Found: [*M*]^+^ 197.0604, [C_10_H_12_ClNO]^+^ requires 197.0607.

## Refinement   

Crystal data, data collection and structure refinement details are summarized in Table 4 ▸. In both **1a** and **1b**, Cl1/Cl1a and C12/C12*a* were modelled as disordered over two positions using restraints (DFIX for C11—C12, C11—C12*a* distances) and constraints (EADP, Cl atoms). The occupancy was allowed to refine with a population parameter of **1a** = 0.783 (2), and **1b** = 0.768 (2). The amide N—H H atom was located in a difference-Fourier map and freely refined. H atoms bonded to carbon were placed with idealized geometry and refined using a riding model with C—H = 0.95 Å aromatic, C—H = 0.90 Å methine, with *U*_iso_(H) = 1.2*U*_eq_(C) and C—H = 0.98 Å methyl with *U*_iso_(H) = 1.5*U*_eq_(C).

## Supplementary Material

Crystal structure: contains datablock(s) . DOI: 10.1107/S2056989018013889/ds2252sup1.cif

Structure factors: contains datablock(s) 1a. DOI: 10.1107/S2056989018013889/ds22521asup2.hkl

Structure factors: contains datablock(s) 1b. DOI: 10.1107/S2056989018013889/ds22521bsup3.hkl

Supporting information file. DOI: 10.1107/S2056989018013889/ds22521asup4.cml

CCDC references: 1870782, 1870781

Additional supporting information:  crystallographic information; 3D view; checkCIF report

## Figures and Tables

**Table 1:** Selected geometric parameters (Å, °) for **1a**, **1b** and IQOHOL

	**1a**	**1b**	IQOHOL^*a*^
Cl1—C11	1.7861 (17)	1.7845 (18)	1.785 (16)
O10—C9	1.2233 (18)	1.2245 (19)	1.219 (15)
N8—C7	1.4226 (19)	1.421 (2)	1.421 (16)
N8—C9	1.3448 (19)	1.344 (2)	1.341 (16)
C9—C11	1.524 (2)	1.523 (2)	1.522 (18)
O10—C9—C11—C12	−60.4 (5)	−60.2 (6)	61.35 (1)
C9—N8—C7—C1	45.3 (2)	45.6 (2)	−44.19 (1)

Note: (*a*) Equivalent geometric parameters are given for IQOHOL as atom labels do not matchthose of **1a** and **1b**.

**Table 2:** Hydrogen-bond geometry (Å, °) for **1a** [Chem scheme1] *Cg*1 is the centroid of the C1–C6 ring.

*D*—H⋯*A*	*D*—H	H⋯*A*	*D*⋯*A*	*D*—H⋯*A*
N8—H8⋯O10^i^	0.80 (2)	2.03 (2)	2.8295 (16)	174.8 (17)
C11—H11⋯O10^i^	1.00	2.48	3.3574 (18)	146
C12—H12*E*⋯*Cg*1^ii^	0.98	2.61	3.503 (11)	151

Symmetry codes: (i) 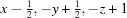; (ii) 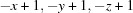.

**Table 3:** Hydrogen-bond geometry (Å, °) for **1b** [Chem scheme1] *Cg*1 is the centroid of the C1–C6 ring.

*D*—H⋯*A*	*D*—H	H⋯*A*	*D*⋯*A*	*D*—H⋯*A*
N8—H8⋯O10^i^	0.83 (2)	2.00 (2)	2.8255 (18)	174.2 (19)
C11—H11⋯O10^i^	1.00	2.48	3.353 (2)	146
C12—H12*E*⋯*Cg*1^ii^	0.98	2.62	3.493 (13)	149

Symmetry codes: (i) 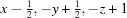; (ii) 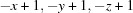.

**Table 4:** Experimental details

	(1a)	(1b)
Crystal data		
Chemical formula	C_10_H_12_ClNO	C_10_H_12_ClNO
*M*_r_	197.66	197.66
Crystal system, space group	Orthorhombic, *P* *b* *c* *a*	Orthorhombic, *P* *b* *c* *a*
Temperature (K)	100	100
*a*, *b*, *c* (Å)	9.5119 (3), 9.6885 (4), 21.8439 (8)	9.5053 (6), 9.6793 (5), 21.8380 (13)
*V* (Å^3^)	2013.05 (13)	2009.2 (2)
*Z*	8	8
Radiation type	Cu *K*α	Mo *K*α
μ (mm^−1^)	3.03	0.34
Crystal size (mm)	0.27 × 0.14 × 0.10	0.25 × 0.11 × 0.1

Data collection		
Diffractometer	Bruker APEXII Kappa Duo	Bruker D8 Quest ECO
Absorption correction	Multi-scan (*SADABS*; Bruker, 2016 ▸)	Multi-scan (*SADABS*; Bruker, 2016 ▸)
*T*_min_, *T*_max_	0.565, 0.753	0.702, 0.745
No. of measured, independent and observed [*I* > 2σ(*I*)] reflections	18191, 1892, 1819	19741, 2061, 1668
*R*_int_	0.045	0.051
(sin θ/λ)_max_ (Å^−1^)	0.608	0.627

Refinement		
*R*[*F* ^2^ > 2σ(*F* ^2^)], *wR*(*F* ^2^), *S*	0.038, 0.103, 1.06	0.037, 0.089, 1.10
No. of reflections	1892	2061
No. of parameters	138	138
No. of restraints	2	2
H-atom treatment	H atoms treated by a mixture of independent and constrained refinement	H atoms treated by a mixture of independent and constrained refinement
Δρ_max_, Δρ_min_ (e Å^−3^)	0.29, −0.26	0.30, −0.31

Computer programs: *APEX3* (Bruker, 2016 ▸), *SAINT* (Bruker, 2015 ▸), *SHELXT* (Sheldrick, 2015*a* ▸), *SHELXL* (Sheldrick, 2015*b* ▸) and *OLEX2* (Dolomanov *et al.*, 2009 ▸).

## supplementary crystallographic information

### 2-Chloro-N-(4-methylphenyl)propanamide (1a) Crystal data

**Table:**

C_10_H_12_ClNO	*D*_x_ = 1.304 Mg m^−^^3^
*M**_r_* = 197.66	Cu *K*α radiation, λ = 1.54178 Å
Orthorhombic, *P**b**c**a*	Cell parameters from 9895 reflections
*a* = 9.5119 (3) Å	θ = 4.1–69.6°
*b* = 9.6885 (4) Å	µ = 3.03 mm^−^^1^
*c* = 21.8439 (8) Å	*T* = 100 K
*V* = 2013.05 (13) Å^3^	Irregular, clear colourless
*Z* = 8	0.27 × 0.14 × 0.10 mm
*F*(000) = 832	

### 2-Chloro-N-(4-methylphenyl)propanamide (1a) Data collection

**Table:**

Bruker APEXII Kappa Duo diffractometer	1892 independent reflections
Radiation source: microfocus sealed X-ray tube, Incoatec Iµs	1819 reflections with *I* > 2σ(*I*)
Mirror optics monochromator	*R*_int_ = 0.045
Detector resolution: 7.9 pixels mm^-1^	θ_max_ = 69.7°, θ_min_ = 4.1°
ω and φ scans	*h* = −11→11
Absorption correction: multi-scan (SADABS; Bruker, 2016)	*k* = −11→11
*T*_min_ = 0.565, *T*_max_ = 0.753	*l* = −26→26
18191 measured reflections	

### 2-Chloro-N-(4-methylphenyl)propanamide (1a) Refinement

**Table:**

Refinement on *F*^2^	Primary atom site location: dual
Least-squares matrix: full	Hydrogen site location: mixed
*R*[*F*^2^ > 2σ(*F*^2^)] = 0.038	H atoms treated by a mixture of independent and constrained refinement
*wR*(*F*^2^) = 0.103	*w* = 1/[σ^2^(*F*_o_^2^) + (0.0541*P*)^2^ + 1.2115*P*] where *P* = (*F*_o_^2^ + 2*F*_c_^2^)/3
*S* = 1.06	(Δ/σ)_max_ < 0.001
1892 reflections	Δρ_max_ = 0.29 e Å^−^^3^
138 parameters	Δρ_min_ = −0.26 e Å^−^^3^
2 restraints	

### 2-Chloro-N-(4-methylphenyl)propanamide (1a) Special details

**Table:**

Geometry. All esds (except the esd in the dihedral angle between two l.s. planes) are estimated using the full covariance matrix. The cell esds are taken into account individually in the estimation of esds in distances, angles and torsion angles; correlations between esds in cell parameters are only used when they are defined by crystal symmetry. An approximate (isotropic) treatment of cell esds is used for estimating esds involving l.s. planes.
Refinement. The terminal chloro/methyl groups are disordered and overlap with an occupancy of 78:22%. The disorder was modelled with restraints (DFIX) and constraints (EADP for the Cl atoms).

### 2-Chloro-N-(4-methylphenyl)propanamide (1a) Fractional atomic coordinates and isotropic or equivalent isotropic displacement parameters (Å^2^)

**Table:**

	*x*	*y*	*z*	*U*_iso_*/*U*_eq_	Occ. (<1)
Cl1	0.60104 (11)	0.01803 (8)	0.42660 (5)	0.0298 (2)	0.783 (2)
Cl1A	0.6006 (11)	0.2847 (11)	0.3649 (4)	0.0328 (12)	0.217 (2)
O10	0.74965 (11)	0.26055 (12)	0.48882 (5)	0.0281 (3)	
N8	0.53542 (13)	0.31060 (13)	0.52846 (6)	0.0208 (3)	
H8	0.453 (2)	0.2949 (17)	0.5247 (7)	0.016 (4)*	
C1	0.69159 (16)	0.46725 (17)	0.58476 (7)	0.0257 (4)	
H1	0.7392	0.4906	0.5480	0.031*	
C2	0.73137 (17)	0.52711 (18)	0.63985 (8)	0.0303 (4)	
H2	0.8067	0.5915	0.6402	0.036*	
C3	0.66349 (18)	0.49513 (17)	0.69457 (8)	0.0286 (4)	
C4	0.7043 (2)	0.5651 (2)	0.75375 (9)	0.0408 (5)	
H4A	0.7236	0.4951	0.7850	0.061*	
H4B	0.6272	0.6246	0.7675	0.061*	
H4C	0.7888	0.6212	0.7471	0.061*	
C5	0.55361 (18)	0.40046 (17)	0.69264 (7)	0.0286 (4)	
H5	0.5058	0.3771	0.7294	0.034*	
C6	0.51263 (16)	0.33958 (16)	0.63802 (7)	0.0253 (3)	
H6	0.4374	0.2751	0.6376	0.030*	
C7	0.58167 (15)	0.37290 (16)	0.58389 (7)	0.0208 (3)	
C9	0.62129 (15)	0.25772 (15)	0.48554 (7)	0.0204 (3)	
C11	0.54665 (16)	0.19425 (16)	0.43058 (7)	0.0223 (3)	
H11	0.4426	0.1988	0.4369	0.027*	0.783 (2)
H11A	0.4428	0.2050	0.4358	0.027*	0.217 (2)
C12A	0.582 (2)	0.0409 (11)	0.4215 (10)	0.035 (2)	0.217 (2)
H12A	0.5437	−0.0127	0.4558	0.053*	0.217 (2)
H12B	0.6838	0.0290	0.4197	0.053*	0.217 (2)
H12C	0.5395	0.0084	0.3831	0.053*	0.217 (2)
C12	0.5856 (12)	0.2688 (11)	0.3711 (4)	0.035 (2)	0.783 (2)
H12D	0.6871	0.2610	0.3641	0.053*	0.783 (2)
H12E	0.5597	0.3664	0.3743	0.053*	0.783 (2)
H12F	0.5350	0.2266	0.3368	0.053*	0.783 (2)

### 2-Chloro-N-(4-methylphenyl)propanamide (1a) Atomic displacement parameters (Å^2^)

**Table:**

	*U*^11^	*U*^22^	*U*^33^	*U*^12^	*U*^13^	*U*^23^
Cl1	0.0401 (5)	0.0204 (4)	0.0290 (4)	0.0037 (2)	−0.0030 (3)	−0.0030 (3)
Cl1A	0.034 (2)	0.036 (2)	0.0278 (15)	−0.0036 (14)	−0.0033 (13)	0.0100 (13)
O10	0.0158 (6)	0.0365 (7)	0.0320 (6)	0.0011 (5)	0.0005 (4)	−0.0073 (5)
N8	0.0125 (6)	0.0255 (7)	0.0244 (6)	−0.0016 (5)	0.0002 (5)	−0.0020 (5)
C1	0.0207 (7)	0.0299 (8)	0.0266 (8)	−0.0025 (6)	0.0027 (6)	−0.0026 (6)
C2	0.0215 (8)	0.0348 (9)	0.0347 (9)	−0.0034 (7)	−0.0014 (7)	−0.0071 (7)
C3	0.0281 (8)	0.0310 (8)	0.0266 (8)	0.0070 (7)	−0.0060 (7)	−0.0037 (6)
C4	0.0409 (10)	0.0500 (11)	0.0315 (9)	0.0061 (9)	−0.0089 (8)	−0.0112 (9)
C5	0.0332 (8)	0.0295 (8)	0.0233 (7)	0.0048 (7)	0.0034 (6)	0.0033 (6)
C6	0.0242 (7)	0.0230 (7)	0.0286 (8)	−0.0005 (6)	0.0032 (6)	0.0016 (6)
C7	0.0181 (7)	0.0215 (7)	0.0229 (7)	0.0032 (6)	−0.0005 (5)	−0.0003 (6)
C9	0.0182 (7)	0.0195 (7)	0.0235 (7)	0.0001 (5)	0.0010 (5)	0.0024 (6)
C11	0.0175 (7)	0.0244 (8)	0.0250 (7)	0.0020 (6)	0.0010 (5)	−0.0016 (6)
C12A	0.034 (3)	0.030 (3)	0.042 (4)	0.006 (2)	−0.007 (2)	−0.005 (2)
C12	0.034 (3)	0.030 (3)	0.042 (4)	0.006 (2)	−0.007 (2)	−0.005 (2)

### 2-Chloro-N-(4-methylphenyl)propanamide (1a) Geometric parameters (Å, º)

**Table:**

Cl1—C11	1.7861 (17)	C5—H5	0.9500
Cl1A—C11	1.758 (8)	C5—C6	1.387 (2)
O10—C9	1.2233 (18)	C6—H6	0.9500
N8—H8	0.80 (2)	C6—C7	1.391 (2)
N8—C7	1.4226 (19)	C9—C11	1.524 (2)
N8—C9	1.3448 (19)	C11—H11	1.0000
C1—H1	0.9500	C11—H11A	1.0000
C1—C2	1.388 (2)	C11—C12A	1.536 (9)
C1—C7	1.389 (2)	C11—C12	1.532 (7)
C2—H2	0.9500	C12A—H12A	0.9800
C2—C3	1.394 (2)	C12A—H12B	0.9800
C3—C4	1.511 (2)	C12A—H12C	0.9800
C3—C5	1.391 (2)	C12—H12D	0.9800
C4—H4A	0.9800	C12—H12E	0.9800
C4—H4B	0.9800	C12—H12F	0.9800
C4—H4C	0.9800		
			
C7—N8—H8	118.0 (12)	O10—C9—N8	123.83 (14)
C9—N8—H8	116.7 (12)	O10—C9—C11	121.34 (13)
C9—N8—C7	124.55 (13)	N8—C9—C11	114.82 (13)
C2—C1—H1	120.3	Cl1—C11—H11	109.6
C2—C1—C7	119.47 (15)	Cl1A—C11—H11A	109.2
C7—C1—H1	120.3	C9—C11—Cl1	106.80 (10)
C1—C2—H2	119.2	C9—C11—Cl1A	107.8 (4)
C1—C2—C3	121.63 (16)	C9—C11—H11	109.6
C3—C2—H2	119.2	C9—C11—H11A	109.2
C2—C3—C4	121.00 (16)	C9—C11—C12A	113.1 (8)
C5—C3—C2	117.95 (15)	C9—C11—C12	111.4 (5)
C5—C3—C4	121.02 (16)	C12A—C11—Cl1A	108.3 (9)
C3—C4—H4A	109.5	C12A—C11—H11A	109.2
C3—C4—H4B	109.5	C12—C11—Cl1	109.8 (4)
C3—C4—H4C	109.5	C12—C11—H11	109.6
H4A—C4—H4B	109.5	C11—C12A—H12A	109.5
H4A—C4—H4C	109.5	C11—C12A—H12B	109.5
H4B—C4—H4C	109.5	C11—C12A—H12C	109.5
C3—C5—H5	119.4	H12A—C12A—H12B	109.5
C6—C5—C3	121.17 (15)	H12A—C12A—H12C	109.5
C6—C5—H5	119.4	H12B—C12A—H12C	109.5
C5—C6—H6	120.0	C11—C12—H12D	109.5
C5—C6—C7	120.01 (15)	C11—C12—H12E	109.5
C7—C6—H6	120.0	C11—C12—H12F	109.5
C1—C7—N8	121.58 (14)	H12D—C12—H12E	109.5
C1—C7—C6	119.77 (14)	H12D—C12—H12F	109.5
C6—C7—N8	118.63 (14)	H12E—C12—H12F	109.5
			
O10—C9—C11—Cl1	59.49 (17)	C2—C1—C7—C6	0.1 (2)
O10—C9—C11—Cl1A	−59.4 (4)	C2—C3—C5—C6	0.0 (2)
O10—C9—C11—C12A	60.2 (10)	C3—C5—C6—C7	0.0 (2)
O10—C9—C11—C12	−60.4 (5)	C4—C3—C5—C6	177.74 (16)
N8—C9—C11—Cl1	−121.40 (12)	C5—C6—C7—N8	−178.85 (14)
N8—C9—C11—Cl1A	119.7 (4)	C5—C6—C7—C1	0.0 (2)
N8—C9—C11—C12A	−120.7 (10)	C7—N8—C9—O10	−1.9 (2)
N8—C9—C11—C12	118.7 (5)	C7—N8—C9—C11	179.00 (13)
C1—C2—C3—C4	−177.66 (16)	C7—C1—C2—C3	−0.1 (3)
C1—C2—C3—C5	0.1 (3)	C9—N8—C7—C1	45.3 (2)
C2—C1—C7—N8	178.89 (14)	C9—N8—C7—C6	−135.93 (15)

### 2-Chloro-N-(4-methylphenyl)propanamide (1a) Hydrogen-bond geometry (Å, º)

Cg1 is the centroid of the C1–C6 ring.

**Table:**

*D*—H···*A*	*D*—H	H···*A*	*D*···*A*	*D*—H···*A*
N8—H8···O10^i^	0.80 (2)	2.03 (2)	2.8295 (16)	174.8 (17)
C11—H11···O10^i^	1.00	2.48	3.3574 (18)	146
C12—H12*E*···*Cg*1^ii^	0.98	2.61	3.503 (11)	151

Symmetry codes: (i) *x*−1/2, −*y*+1/2, −*z*+1; (ii) −*x*+1, −*y*+1, −*z*+1.

### (1b) Crystal data

**Table:**

C_10_H_12_ClNO	*D*_x_ = 1.307 Mg m^−^^3^
*M**_r_* = 197.66	Mo *K*α radiation, λ = 0.71073 Å
Orthorhombic, *P**b**c**a*	Cell parameters from 6677 reflections
*a* = 9.5053 (6) Å	θ = 2.8–26.5°
*b* = 9.6793 (5) Å	µ = 0.34 mm^−^^1^
*c* = 21.8380 (13) Å	*T* = 100 K
*V* = 2009.2 (2) Å^3^	Fragment, clear colourless
*Z* = 8	0.25 × 0.11 × 0.1 mm
*F*(000) = 832	

### (1b) Data collection

**Table:**

Bruker D8 Quest ECO diffractometer	2061 independent reflections
Radiation source: sealed X-ray tube, Siemens, KFF Mo 2K -90 C	1668 reflections with *I* > 2σ(*I*)
Graphite monochromator	*R*_int_ = 0.051
Detector resolution: 5.12 pixels mm^-1^	θ_max_ = 26.5°, θ_min_ = 3.5°
ω and φ scans	*h* = −11→9
Absorption correction: multi-scan (SADABS; Bruker, 2016)	*k* = −12→12
*T*_min_ = 0.702, *T*_max_ = 0.745	*l* = −26→27
19741 measured reflections	

### (1b) Refinement

**Table:**

Refinement on *F*^2^	Primary atom site location: dual
Least-squares matrix: full	Hydrogen site location: mixed
*R*[*F*^2^ > 2σ(*F*^2^)] = 0.037	H atoms treated by a mixture of independent and constrained refinement
*wR*(*F*^2^) = 0.089	*w* = 1/[σ^2^(*F*_o_^2^) + (0.0322*P*)^2^ + 1.4949*P*] where *P* = (*F*_o_^2^ + 2*F*_c_^2^)/3
*S* = 1.10	(Δ/σ)_max_ < 0.001
2061 reflections	Δρ_max_ = 0.30 e Å^−^^3^
138 parameters	Δρ_min_ = −0.31 e Å^−^^3^
2 restraints	

### (1b) Special details

**Table:**

Geometry. All esds (except the esd in the dihedral angle between two l.s. planes) are estimated using the full covariance matrix. The cell esds are taken into account individually in the estimation of esds in distances, angles and torsion angles; correlations between esds in cell parameters are only used when they are defined by crystal symmetry. An approximate (isotropic) treatment of cell esds is used for estimating esds involving l.s. planes.
Refinement. The terminal chloro/methyl groups are disordered and overlap with an occupancy of 77:23%. The disorder was modelled with restraints (DFIX) and constraints (EADP for the Cl atoms).

### (1b) Fractional atomic coordinates and isotropic or equivalent isotropic displacement parameters (Å^2^)

**Table:**

	*x*	*y*	*z*	*U*_iso_*/*U*_eq_	Occ. (<1)
Cl1	0.60085 (14)	0.01841 (9)	0.42661 (6)	0.0233 (2)	0.768 (2)
Cl1A	0.5999 (11)	0.2831 (11)	0.3648 (4)	0.0253 (12)	0.232 (2)
O10	0.74993 (12)	0.26099 (13)	0.48878 (5)	0.0217 (3)	
N8	0.53537 (14)	0.31069 (14)	0.52838 (6)	0.0141 (3)	
H8	0.450 (2)	0.2946 (19)	0.5248 (9)	0.019 (5)*	
C1	0.69140 (18)	0.46745 (18)	0.58487 (8)	0.0186 (4)	
H1	0.7389	0.4913	0.5481	0.022*	
C2	0.73115 (18)	0.52705 (19)	0.63992 (8)	0.0236 (4)	
H2	0.8064	0.5916	0.6404	0.028*	
C3	0.66346 (19)	0.49466 (19)	0.69465 (8)	0.0217 (4)	
C4	0.7041 (2)	0.5647 (2)	0.75373 (9)	0.0331 (5)	
H4A	0.6236	0.6170	0.7695	0.050*	
H4B	0.7829	0.6278	0.7462	0.050*	
H4C	0.7323	0.4949	0.7838	0.050*	
C5	0.5537 (2)	0.39994 (18)	0.69253 (8)	0.0220 (4)	
H5	0.5059	0.3763	0.7293	0.026*	
C6	0.51244 (18)	0.33931 (18)	0.63806 (8)	0.0187 (4)	
H6	0.4370	0.2750	0.6376	0.022*	
C7	0.58171 (16)	0.37265 (16)	0.58381 (7)	0.0140 (3)	
C9	0.62137 (16)	0.25791 (16)	0.48549 (7)	0.0135 (3)	
C11	0.54663 (17)	0.19470 (17)	0.43051 (8)	0.0158 (3)	
H11	0.4425	0.1995	0.4367	0.019*	0.768 (2)
H11A	0.4426	0.2044	0.4357	0.019*	0.232 (2)
C12A	0.585 (3)	0.0413 (12)	0.4233 (12)	0.033 (3)	0.232 (2)
H12A	0.5527	−0.0098	0.4595	0.049*	0.232 (2)
H12B	0.6867	0.0317	0.4191	0.049*	0.232 (2)
H12C	0.5383	0.0041	0.3867	0.049*	0.232 (2)
C12	0.5869 (14)	0.2707 (13)	0.3713 (4)	0.033 (3)	0.768 (2)
H12D	0.6883	0.2615	0.3643	0.049*	0.768 (2)
H12E	0.5625	0.3687	0.3752	0.049*	0.768 (2)
H12F	0.5355	0.2303	0.3368	0.049*	0.768 (2)

### (1b) Atomic displacement parameters (Å^2^)

**Table:**

	*U*^11^	*U*^22^	*U*^33^	*U*^12^	*U*^13^	*U*^23^
Cl1	0.0348 (6)	0.0136 (3)	0.0214 (4)	0.0023 (3)	−0.0026 (3)	−0.0033 (3)
Cl1A	0.032 (2)	0.027 (2)	0.0167 (16)	0.0008 (15)	−0.0042 (14)	0.0061 (13)
O10	0.0104 (6)	0.0306 (7)	0.0242 (7)	0.0017 (5)	0.0004 (5)	−0.0076 (5)
N8	0.0078 (7)	0.0183 (7)	0.0161 (7)	−0.0014 (5)	−0.0005 (6)	−0.0017 (6)
C1	0.0156 (8)	0.0216 (9)	0.0185 (9)	−0.0030 (7)	0.0031 (6)	−0.0029 (7)
C2	0.0166 (9)	0.0273 (10)	0.0268 (10)	−0.0046 (7)	−0.0006 (7)	−0.0077 (8)
C3	0.0216 (9)	0.0238 (9)	0.0196 (9)	0.0063 (7)	−0.0061 (7)	−0.0040 (7)
C4	0.0351 (11)	0.0407 (12)	0.0234 (10)	0.0051 (9)	−0.0089 (8)	−0.0098 (9)
C5	0.0293 (10)	0.0222 (9)	0.0145 (8)	0.0042 (7)	0.0041 (7)	0.0040 (7)
C6	0.0195 (8)	0.0166 (8)	0.0201 (9)	−0.0017 (7)	0.0029 (7)	0.0015 (7)
C7	0.0130 (8)	0.0144 (8)	0.0145 (8)	0.0023 (6)	0.0001 (6)	−0.0001 (6)
C9	0.0124 (8)	0.0131 (7)	0.0151 (8)	0.0007 (6)	0.0012 (6)	0.0014 (6)
C11	0.0125 (7)	0.0179 (8)	0.0169 (8)	0.0009 (6)	0.0018 (6)	−0.0017 (7)
C12A	0.028 (3)	0.031 (4)	0.039 (5)	0.009 (3)	−0.007 (3)	−0.006 (3)
C12	0.028 (3)	0.031 (4)	0.039 (5)	0.009 (3)	−0.007 (3)	−0.006 (3)

### (1b) Geometric parameters (Å, º)

**Table:**

Cl1—C11	1.7845 (18)	C5—H5	0.9500
Cl1A—C11	1.746 (8)	C5—C6	1.383 (2)
O10—C9	1.2245 (19)	C6—H6	0.9500
N8—H8	0.83 (2)	C6—C7	1.393 (2)
N8—C7	1.421 (2)	C9—C11	1.523 (2)
N8—C9	1.344 (2)	C11—H11	1.0000
C1—H1	0.9500	C11—H11A	1.0000
C1—C2	1.386 (2)	C11—C12A	1.536 (9)
C1—C7	1.389 (2)	C11—C12	1.535 (7)
C2—H2	0.9500	C12A—H12A	0.9800
C2—C3	1.393 (3)	C12A—H12B	0.9800
C3—C4	1.508 (2)	C12A—H12C	0.9800
C3—C5	1.390 (3)	C12—H12D	0.9800
C4—H4A	0.9800	C12—H12E	0.9800
C4—H4B	0.9800	C12—H12F	0.9800
C4—H4C	0.9800		
			
C7—N8—H8	117.6 (13)	O10—C9—N8	123.83 (15)
C9—N8—H8	117.2 (14)	O10—C9—C11	121.43 (14)
C9—N8—C7	124.44 (14)	N8—C9—C11	114.73 (14)
C2—C1—H1	120.2	Cl1—C11—H11	109.7
C2—C1—C7	119.60 (16)	Cl1A—C11—H11A	109.5
C7—C1—H1	120.2	C9—C11—Cl1	106.68 (12)
C1—C2—H2	119.2	C9—C11—Cl1A	108.4 (4)
C1—C2—C3	121.64 (17)	C9—C11—H11	109.7
C3—C2—H2	119.2	C9—C11—H11A	109.5
C2—C3—C4	120.96 (17)	C9—C11—C12A	111.1 (9)
C5—C3—C2	117.83 (16)	C9—C11—C12	110.8 (5)
C5—C3—C4	121.17 (17)	C12A—C11—Cl1A	108.8 (10)
C3—C4—H4A	109.5	C12A—C11—H11A	109.5
C3—C4—H4B	109.5	C12—C11—Cl1	110.2 (5)
C3—C4—H4C	109.5	C12—C11—H11	109.7
H4A—C4—H4B	109.5	C11—C12A—H12A	109.5
H4A—C4—H4C	109.5	C11—C12A—H12B	109.5
H4B—C4—H4C	109.5	C11—C12A—H12C	109.5
C3—C5—H5	119.3	H12A—C12A—H12B	109.5
C6—C5—C3	121.41 (16)	H12A—C12A—H12C	109.5
C6—C5—H5	119.3	H12B—C12A—H12C	109.5
C5—C6—H6	120.0	C11—C12—H12D	109.5
C5—C6—C7	119.94 (16)	C11—C12—H12E	109.5
C7—C6—H6	120.0	C11—C12—H12F	109.5
C1—C7—N8	121.71 (14)	H12D—C12—H12E	109.5
C1—C7—C6	119.58 (15)	H12D—C12—H12F	109.5
C6—C7—N8	118.69 (14)	H12E—C12—H12F	109.5
			
O10—C9—C11—Cl1	59.75 (18)	C2—C1—C7—C6	0.4 (2)
O10—C9—C11—Cl1A	−58.9 (4)	C2—C3—C5—C6	0.1 (3)
O10—C9—C11—C12A	60.5 (11)	C3—C5—C6—C7	0.2 (3)
O10—C9—C11—C12	−60.2 (6)	C4—C3—C5—C6	177.51 (17)
N8—C9—C11—Cl1	−121.32 (14)	C5—C6—C7—N8	−178.85 (15)
N8—C9—C11—Cl1A	120.0 (4)	C5—C6—C7—C1	−0.4 (2)
N8—C9—C11—C12A	−120.6 (11)	C7—N8—C9—O10	−2.1 (3)
N8—C9—C11—C12	118.7 (6)	C7—N8—C9—C11	178.97 (14)
C1—C2—C3—C4	−177.50 (17)	C7—C1—C2—C3	−0.2 (3)
C1—C2—C3—C5	0.0 (3)	C9—N8—C7—C1	45.6 (2)
C2—C1—C7—N8	178.82 (16)	C9—N8—C7—C6	−136.01 (17)

### (1b) Hydrogen-bond geometry (Å, º)

Cg1 is the centroid of the C1–C6 ring.

**Table:**

*D*—H···*A*	*D*—H	H···*A*	*D*···*A*	*D*—H···*A*
N8—H8···O10^i^	0.83 (2)	2.00 (2)	2.8255 (18)	174.2 (19)
C11—H11···O10^i^	1.00	2.48	3.353 (2)	146
C12—H12*E*···*Cg*1^ii^	0.98	2.62	3.493 (13)	149

Symmetry codes: (i) *x*−1/2, −*y*+1/2, −*z*+1; (ii) −*x*+1, −*y*+1, −*z*+1.
